# Multiple mechanisms of response suppression to self-induced sensation during pursuit eye movements

**DOI:** 10.1098/rsos.250967

**Published:** 2025-10-29

**Authors:** Omar Bachtoula, Mel Ellul Miraval, Ignacio Serrano-Pedraza, David Souto

**Affiliations:** ^1^ Department of Experimental Psychology, Complutense University of Madrid, Madrid, Spain; ^2^ School of Psychology and Vision Sciences, University of Leicester, Leicester, UK

**Keywords:** optokinetic nystagmus, smooth pursuit eye movements, reafference, optokinesis, motor suppression, sensory attenuation

## Abstract

Eye movements generate a perceptual challenge, that of distinguishing self-induced sensations from movement in the world. We ask about the mechanisms involved in suppressing eye movements towards self-induced sensation, ensuring visual stability. When tracking with the eyes an object moving against a textured background, the background retinal image moves in the opposite direction to the smooth pursuit eye movement. Optokinetic responses, such as optokinetic nystagmus or ocular tracking to this reafferent signal, must be suppressed to sustain the pursuit of the object of interest. We varied the contrast of a brief background motion signal to tell apart two plausible accounts of the suppression of optokinesis during pursuit; a visuomotor gain modulation account, which predicts that ocular tracking of background motion is suppressed in the same proportion irrespective of contrast, and a sensory attenuation account, which predicts that larger contrasts are needed to elicit the same response. Unexpectedly, neither account fit ocular tracking in the reafferent signal direction. The combination of contrast-dependent gating, with maximal suppression observed with higher contrasts, and either contrast gain or visuomotor gain modulation, provides a good fit for most observers’ data. Contrast-dependent gating promotes visuomotor stability in response to most salient signals, as a likely adaptation to the statistics of the environment.

## 1. Introduction

In sensory systems, self-induced sensation, also known as reafference, must be accounted for to accurately signal external events [1,2]. A corollary discharge, a copy of the motor command conveying information about the impending movement to sensory brain areas [3], could allow the recovery of external signals by discounting expected sensory consequences of movements. Corollary discharges are believed to play a role in maintaining perceptual continuity during saccades [4], which can be achieved by different forms of downregulation of self-induced sensation [5,6] (but see [7]). During saccades, self-generated retinal motion and location changes are strongly attenuated by visual masking and other active mechanisms [5,8,9], contributing to the impression of visual stability despite discontinuous sensory input. In contrast, during smooth pursuit eye movements (pursuit for short), whereas a reflexive response to reafferent motion needs cancelling, it is less clear whether the sensory processing of reafferent signals is attenuated.

Pursuit eye movements, which aim to match a moving object’s velocity (e.g. a car), generate self-induced retinal motion from a textured background (e.g. a traffic scene). During pursuit, the integration of self-induced sensation, that is, the reafferent retinal flow and eye movement speed estimates derived from a corollary discharge can be used to generate stable space-centric representations [10–12]. This integration is nonetheless subject to illusory percepts, such as in the Filehne illusion, in which the static background is seen as moving opposite to the pursuit [13]. These illusions indicate an underestimation of reafference but not necessarily attenuation, as they can result from biased extra-retinal signals [14,15]. On the other hand, motoric responses to background retinal motion need suppressing, since this reafferent signal is an ideal stimulus for optokinetic eye movements, such as optokinetic nystagmus or ocular tracking, which conflicts with the ongoing voluntary pursuit eye movement.

Suppression of optokinesis during pursuit may take several forms, including the attenuation of self-generated sensation [16]. Several paradigms have been used to investigate the suppression of optokinesis during pursuit, such as by eliciting adaptation after-effects or by eliciting a reflexive response to sudden changes in the movement of the background [17,18]. The latter paradigm is well-suited to test the contribution of sensory and pre-motor stages involved in the suppression of optokinesis.

**Figure 1.**
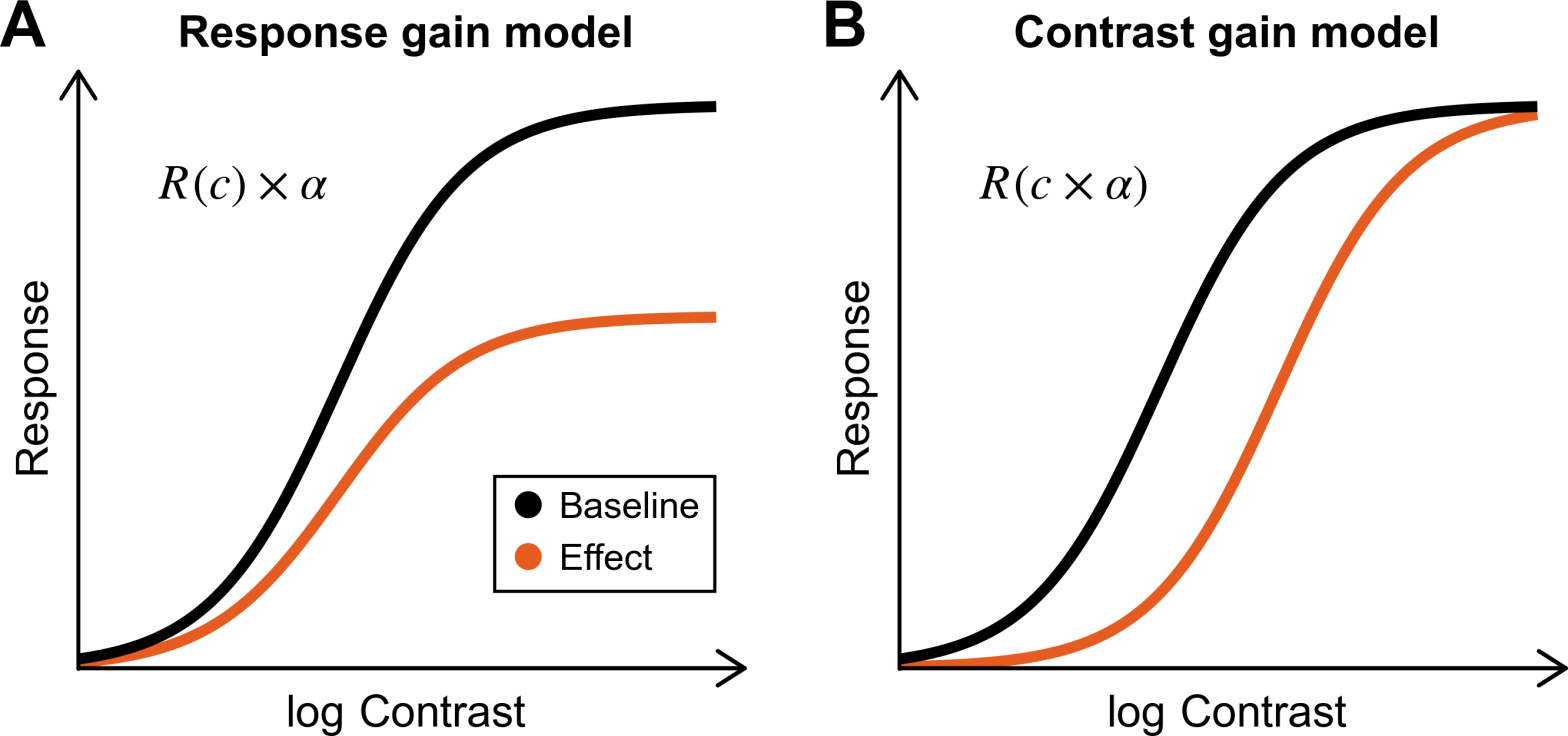
Illustrations of two fundamentally different ways in which a response could be suppressed. (A) The response gain model refers to the amplification of a response. Amplifying the output of some neural process is equivalent to changing the asymptote (in the Naka–Rushton model) of the contrast–response function. (B) The contrast gain model amounts to changing the input. Bringing down the input contrast is equivalent to shifting the contrast–response function rightwards. We hypothesized that the suppression of optokinesis could be achieved mostly through response gain modulation, with little sensory attenuation of the input (rightwards shift).

Many neural responses, such as the firing rates of visual neurons [19] or the amplitude of ocular tracking towards moving gratings [20], increase with visual contrast or signal strength (e.g. proportion of coherent motion signals [16]) in a way that can be fit by the Naka–Rushton model [16]. This functional model, as illustrated in figure 1, specifies the level at which responses asymptote, the rate at which they increase and at which contrasts they increase. The model also makes clear predictions regarding the effects of sensory suppression and visuomotor suppression of optokinesis. If suppression occurs at the level of the response (response gain model), we should have a proportional suppression in the amplitude at every signal level and, as a result, an increased maximal asymptotic response, as shown in figure 1A. Response gain modulation would be consistent with the visuomotor gain modulation proposed [21,22] to account for enhanced ocular responses to target trajectory perturbations during pursuit relative to fixation, where responses to visual motion are amplified to allow accurate pursuit in the presence of smaller retinal motion signals [23]. Response gain modulation could also be responsible for the suppression of optokinesis observed in several studies and paradigms [17,18,24–26].

On the other hand, if suppression occurs at the level of the input (contrast gain modulation), we should see a rightward shift in the contrast–response function (figure 1B)*,* as higher contrasts are needed to elicit the same response. This could be the case if, for instance, a signal originating in pre-motor areas selectively inhibits neurons that process motion in the reafferent direction and drive pursuit, such as those found in the middle superior temporal area (MST), known to combine retinal and extra-retinal pursuit signals [27]. There are also reports consistent with sensory suppression for motion in the reafferent direction during pursuit, such as reports regarding lower contrast sensitivity [28] or suppressed motion blur—accounted for by a sharpening of the temporal impulse response function—in the reafferent direction [29,30].

To tell whether optokinetic response during pursuit fits a response gain, sensory attenuation (a.k.a. contrast gain) or both accounts, we measured ocular tracking response to a background motion perturbation of varying contrasts. Strikingly, we found that the Naka–Rushton function does not provide a full description of oculomotor responses to motion in the reafferent direction. The most parsimonious model requires an extra parameter, corresponding to a contrast-dependent gating mechanism that would selectively cancel responses to high contrast signals.

## 2. Methods

### 2.1. Participants

Participants (*n* = 12, 19−42 years old, seven females) were undergraduate and postgraduate students from the School of Psychology and Vision Sciences, University of Leicester, and three authors. Students were naive to the purpose of the experiment and received vouchers for their participation of a value of £10 per hour.

### 2.2. Materials

A cathode ray tube (CRT) screen was used (Hewlett-Packard P1130, 1280 pixels × 1024 pixels, 85 Hz) to display stimuli 60 cm from the participant. The minimum and maximum luminance of the display were 0.03 and 92.01 cd m^−2^. We used a chinrest to keep the head in place. The right eye (pupil centroid) was tracked by using a video-based eye-tracker (Eyelink 1000, SR Research Ltd, Osgoode, Ontario, Canada) at 1000 Hz. The stimuli were generated by using the Psychophysics toolbox PB-3 for MATLAB [31,32]. The stimuli were gamma-corrected and displayed in greyscale at a 16 bit depth by using a DATAPixx Lite video processor (VPixx Technologies Inc., Saint-Bruno, Canada).

### 2.3. Paradigm and procedure

**Figure 2.**
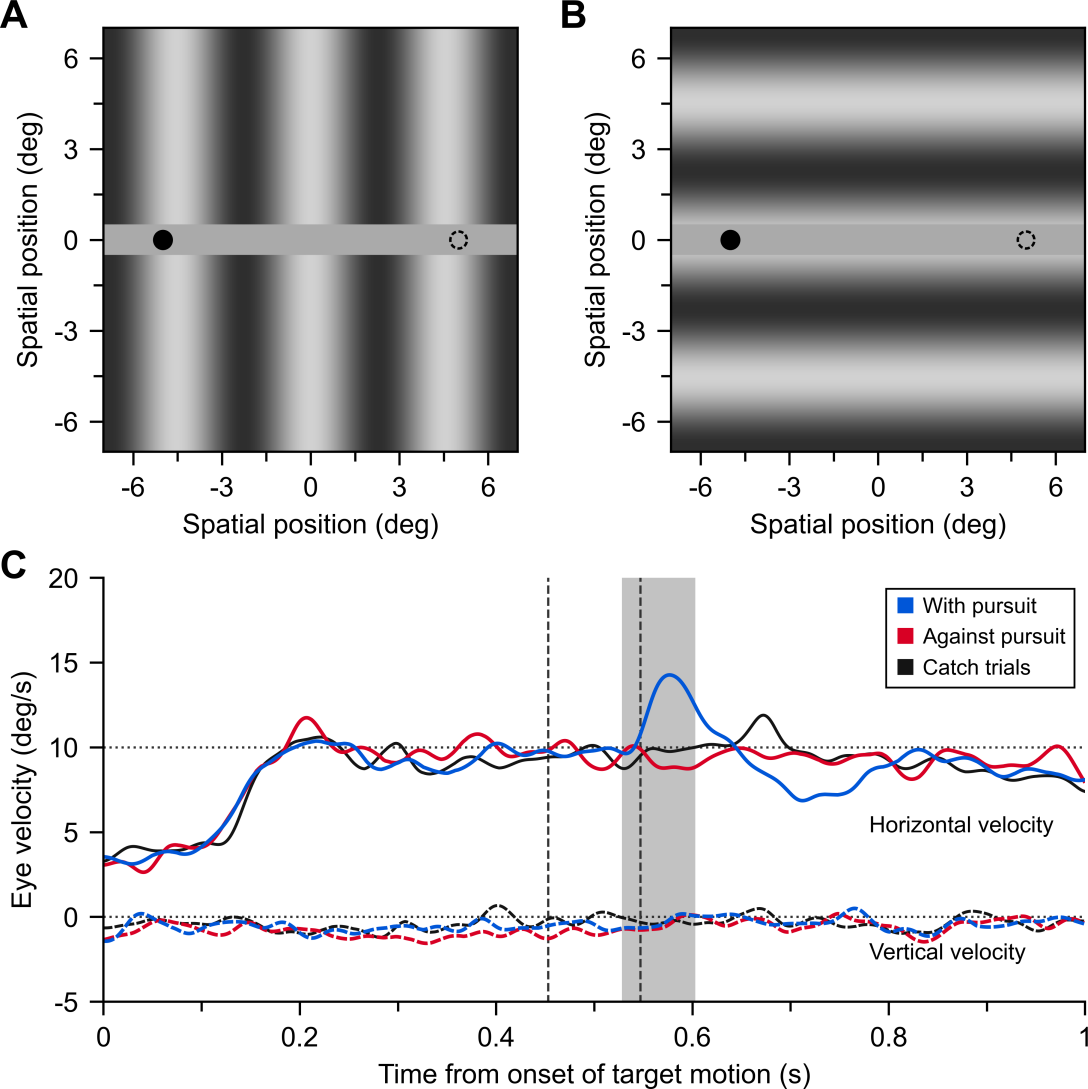
Example of the stimulus and data from the experiment. (A) Example stimulus with a vertical background, where the target (black dot) appears on the left side of the screen. It moves for 1 s at 10 deg s^−1^ to the location indicated by the dashed circle. (B) Example stimulus with a horizontal background. (C) Mean horizontal and vertical eye velocities of participant 11 to perturbations with and against pursuit, plus their corresponding catch trials. The contrast of the background grating was 4% (note that this contrast is lower than the ones shown in panels A and B). The vertical dashed lines indicate the temporal interval where the rapid background perturbation was introduced, and the shaded area corresponds to the interval that we considered for the analysis of the ocular tracking responses.

To probe suppression of optokinesis, we measured ocular responses in the direction of a brief and fast motion in the background during a pursuit eye movement [17,18,24–26]. We selected stimulation parameters that maximize responses to the background [24,33]. As shown in figure 2A,B, the background consisted of low spatial frequency gratings (0.22 cycles per degree (cpd)) oriented either vertically or horizontally, which covered the entire surface of the screen (32.08 deg × 25.82 deg), except for a narrow (1.03 deg) horizontal band (mid-grey, with a luminance of 46.19 cd m^−2^) in the centre of the screen, over which the pursuit target moved horizontally. The background grating had one of seven contrast levels (1%, 2%, 4%, 8%, 16%, 32% or 64%) and its phase was randomized on each trial between −π and π radians. A fixation dot (0.5 deg) stayed on the screen for 0.5 s at 5 deg either left or right off the screen centre. Then the dot moved horizontally to the opposite side at a constant speed (10 deg s^−1^) for 1 s. The participant’s task was to fixate and pursue the dot as accurately as possible while ignoring events occurring in the background. The target direction (leftward if starting from the left-hand side of the screen, rightward otherwise) was randomized within blocks. In some trials, the background drifted for a brief time (94 ms) at a high speed (34 deg s^−1^) in the middle of the target trajectory. This background motion occurred in the interval from 447 to 541 ms after the onset of the target motion (interval between dashed lines in figure 2C). The motion of the background could be upwards or downwards with the horizontal grating, and in the direction of pursuit (‘With pursuit’ condition) or opposite to the direction of pursuit (‘Against pursuit’ condition) with the vertical grating. There were catch trials for every background orientation and contrast, during which there was no motion of the background.

We had then seven contrasts by six conditions (‘Upwards’, ‘Downwards’, ‘Against pursuit’, ‘With Pursuit’, ‘Catch horizontal’ and ‘Catch vertical’). We had five sessions (252 trials), each with six repetitions per condition, leading to a total of 30 repetitions per condition across sessions (1260 trials).

Movies, electronic supplementary materials, S1–S4 (https://figshare.com/s/71391fc3a57e14e7cee3) illustrate the stimulation in four conditions (‘Upwards’, ‘Downwards’, ‘Against pursuit’ and ‘With pursuit’).

### 2.4. Data analysis

Saccades and blinks were detected by using the Eyelink parser, using a 22 deg s^−1^ velocity and a 5000 deg s^−22^ acceleration threshold. This threshold was applied above a velocity baseline measured over the preceding 40 ms, up to 60 deg s^−1^, to account for the ongoing pursuit eye movement. For further processing, we derived eye movement velocity by two-point differentiation of the eye position record, using a 20 ms step [34]. This signal was further filtered by applying a second-order low-pass Butterworth filter with a 20 Hz cut-off.

Figure 2C shows the mean horizontal and vertical eye velocities of one participant in response to the background motion in conditions ‘With pursuit’, ‘Against pursuit’ and catch trials. We plot here the average of de-saccaded and linearly interpolated velocity traces. For this purpose, we extended the Eyelink definition of saccade onsets and offsets by 30 ms based on visual inspection of velocity profiles. Our main analysis concerns a short period of time after background motion. This window is deliberately narrow [33], within 75–150 ms post-motion onset (shaded interval in figure 2C), such that we can average pursuit eye movements uncontaminated by catch-up saccades. We then average data in trials when no saccade was executed during this time period. We obtained a percentage of valid trials between 54% and 100% (*M* = 87%, s.d. = 13%) across participants, based on the above criteria. To estimate the strength of the ocular tracking response, we subtracted the catch trials’ velocity in the corresponding condition (a combination of contrast and background grating orientation) from the average velocity in the presence of background motion. We express responses in a reference frame that is relative to the direction of the background motion. Positive responses indicate an acceleration in the direction of the background motion, and negative responses a deceleration, relative to catch trials.

To fit the data, we used the Naka–Rushton function, as plotted in figure 1A,B,


(2.1) $$R\left(c\right)=R_{max}\frac{c^{n}}{c^{n}+c_{50}^{n}}.$$

In equation (2.1), *R*_max_ is the maximum response without suppression, *c*_50_ is the contrast at half the maximum response and *n* controls the steepness of the transition between minimum and maximum responses.

## 3. Results

We probed the suppression of optokinesis during pursuit eye movements by analysing reflexive responses to a brief background motion perturbation. Figure 2C shows average (de-saccaded and interpolated) velocity traces in one exemplary participant in different conditions. In this example, the pursuit target moves rightward, and a vertical background grating moves briefly (temporal interval indicated by dashed lines) either in the direction of the target (‘With pursuit’, blue line), the opposite direction (‘Against pursuit’, red line) or it does not move (‘Catch trial’, black line). The background grating contrast is 4% in these examples. We can see that, relative to the eye movement velocity in the ‘catch’ trials, after introducing the background perturbation there is a deceleration of horizontal pursuit eye movements (reaching a velocity of about 8 deg s^−1^) in the ‘Against pursuit’ condition, but a much larger acceleration is observed in the ‘With pursuit’ condition, reaching a velocity of about 15 deg s^−1^.

To better quantify the magnitude of the response in each condition (a combination of background motion direction and contrast), we calculated the average eye velocity over a 75−150 ms interval after applying the background perturbation. Then, we subtracted their respective catch trials average (the same combination of background contrast and orientation) and expressed the response direction relative to background motion, with positive values representing a change in velocity in the direction of the background motion.

**Figure 3.**
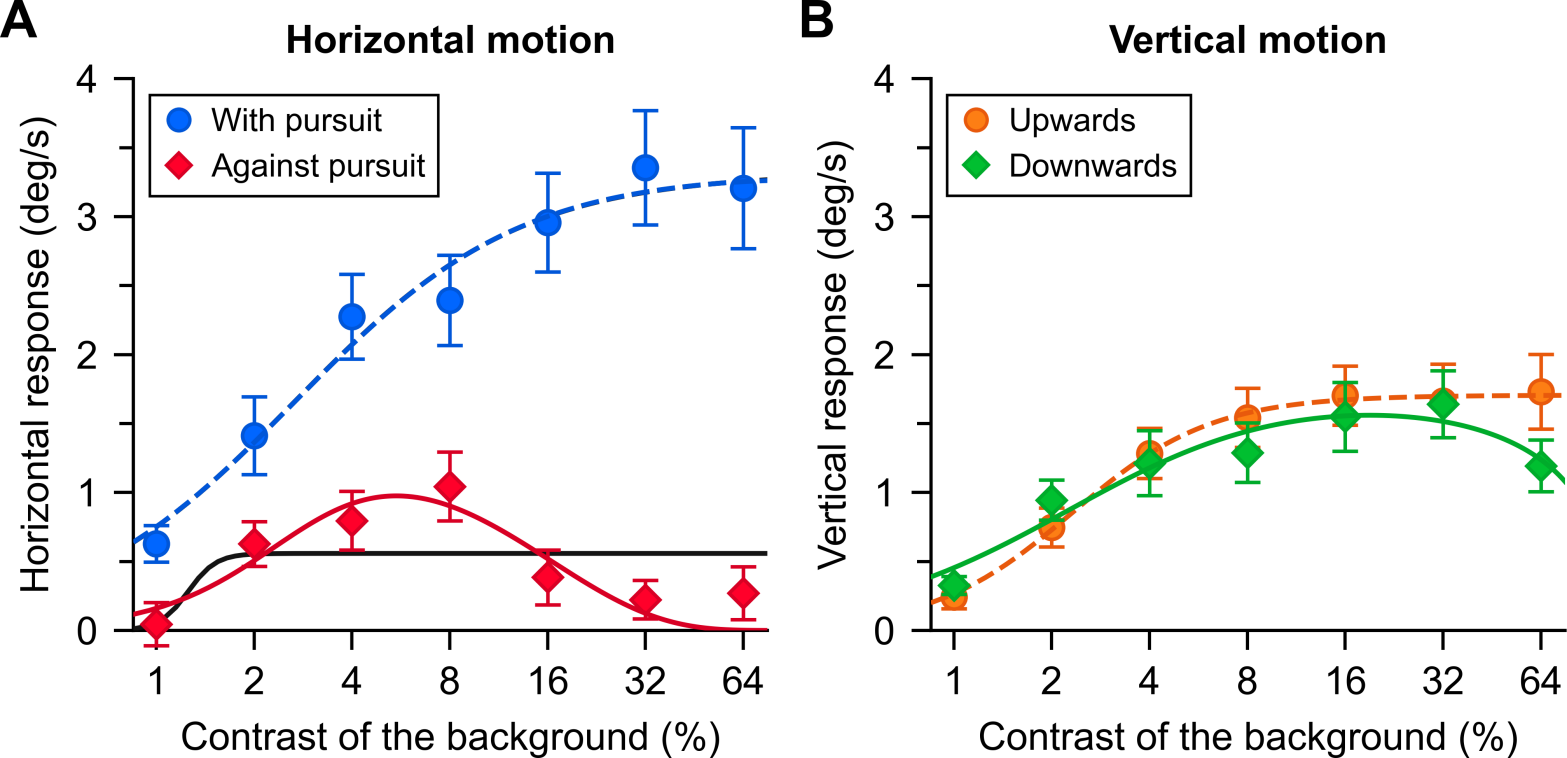
Group average (N = 12) eye movement responses in the direction of background motion for the four perturbation directions as a function of the background contrast. (A) Responses in the direction of pursuit (blue) and responses opposite to the pursuit direction (red). The solid black line shows the Naka–Rushton fit, whereas the solid red line and the dashed blue line show the fits of the gating model. (B) Group average vertical eye movement responses to vertical motion and the fits of the gating model. The fitting procedure was applied to all individual data points (all participants at each contrast), rather than to the means of all participants at each contrast. For clarity, those individual data points are not shown in the figure.

Figure 3 shows the group averages (mean ± s.e.m.) of the mean response to each perturbation direction as a function of the background’s contrast level. Figure 3A contains horizontal eye responses to horizontal perturbations. When the perturbation occurred in the direction of pursuit, the horizontal eye response increased with increasing contrast levels, then saturated at about 16% contrast. Perturbations opposite to the pursuit direction (‘Against pursuit’), however, produced a very different response pattern. As expected, we have suppression, but we also see that in addition to being consistent with response gain modulation (a change in *R*_max_), the data show an unexpected decrease with higher contrasts. Instead of the expected saturation, average responses decreased at 16% contrast to be virtually null at the highest contrasts. These results suggest that response in the reafferent direction does not follow the expected pattern and additional mechanisms, other than contrast or response gain modulation, as modelled by the Naka–Rushton function (black line), are necessary to fit the data.

To assess the statistical significance of these results, we performed a two-way, repeated measures ANOVA for the horizontal motion conditions (‘With pursuit’ and ‘Against pursuit’), with factors ‘Perturbation direction’ and ‘Contrast’. Mauchly’s test indicated that the assumption of sphericity was not met for the main effect of contrast (*W* = 0.006, *p* = 0.002) and the interaction between perturbation direction and contrast (*W* = 0.007, *p* = 0.003), so we are reporting the *F* statistics with adjusted degrees of freedom following the Greenhouse–Geisser estimates of sphericity. The analysis revealed a statistically significant effect of Perturbation direction (*F*(1,11) = 59.12, *p* < 0.001), Contrast (*F*(2.49, 27.34) = 27.21, *p* < 0.001) and an interaction between factors (*F*(3.33, 36.59) = 9.28, *p* < 0.001), indicating that contrast had a different effect on the ocular responses depending on whether the perturbation direction was ‘With pursuit’ or ‘Against pursuit’.

The interaction can be explained by suppressed responses to higher contrasts in the ‘Against pursuit’ condition but not the ‘With pursuit’ condition. Importantly, to confirm the decrease of responses with contrast in the ‘Against pursuit’ condition, we compared responses to the 8% contrast condition with the responses to higher contrasts. The tests revealed statistically significant differences between 8% and 16% contrast conditions (*t*(11) = 2.95, *p* = 0.013, α = 0.05/3 = 0.017; Bonferroni correction), between 8% and 32% contrast conditions (*t*(11) = 3.11, *p* = 0.01, α = 0.017) and between 8% and 64% contrast conditions (*t*(11) = 5.81, *p* < 0.001, α = 0.017).

Figure 3B shows the vertical eye response to the vertical background perturbations. As expected, responses increased with contrast, saturating at about 8% contrast. Both types of vertical perturbation produced similar responses on each contrast level. Once again, we performed a two-way, repeated measures ANOVA with factors ‘Perturbation direction’ and ‘Contrast’. Mauchly’s test indicated that the assumption of sphericity was not met for the factor Contrast (*W* = 0.002, *p* < 0.001). Results revealed a statistically significant effect of Contrast (*F*(1.86, 20.41) = 32.03, *p* < 0.001), and the interaction between both factors (*F*(6, 66) = 2.79, *p* = 0.018), indicating that the ocular responses, as a function of contrast, were different for upwards and downwards background perturbations. However, pairwise comparisons on each contrast level between both types of perturbation did not reach statistical significance when corrected for multiple comparisons with the Bonferroni method (significance level for seven comparisons: α = 0.05/7 = 0.007).

There is a striking difference between these vertical responses, that is, responses orthogonal to the pursuit direction and horizontal responses in the direction of pursuit. Besides seeing that both, vertical responses and horizontal responses in the direction of pursuit, conform to the Naka–Rushton model, we observe that responses in the direction of pursuit reach much higher maximal values (‘With pursuit’ *R*_max_: 1.33−5.54 deg s^−1^; average *R*_max_ of vertical directions: 0.81−3.03 deg s^−1^). The half-saturation parameter, *c*_50_, is harder to estimate for responses against pursuit, as the function is much flatter and is therefore quite variable. The difference between vertical responses and responses in the direction of pursuit could be explained mostly by response gain modulation, as suggested before [23].

To assess whether response gain, sensory attenuation or both explain the suppression of optokinesis in the reafferent direction, we fit a new model to the data that can accommodate the suppression observed in the ‘Against pursuit’ condition. The black lines in figure 3A show the fit of the Naka–Rushton function. Seeing that this function could not account for the responses to perturbations opposite to the pursuit (figure 3A), we fit a modified model in which there is an additional gating mechanism pinning down responses at higher contrasts,


(3.1) $$\;R\left(c\right)=R_{max}\frac{c^{n}}{c^{n}+c_{50}^{n}}{\left(1-c\right)}^{s}.$$

Equation (3.1) involves only the addition of one extra parameter to the Naka–Rushton function, *s*, controlling the intensity of the suppression at high contrasts. Because we observe complete suppression at the highest contrasts in the ‘Against pursuit’ condition, we refer to it as a *gating* mechanism. The parameters of these two models (Naka–Rushton and gating) were constrained as follows: *R*_max_ was limited to the interval [0, max{*R*_With_}], where max{*R*_With_} is the maximum response in the condition ‘With pursuit’, the strongest observed in the data, *c*_50_ was limited to [0,1], *n* was limited to [0,10] and *s* (only in the gating model) was limited to *s* ≥ 0.

Our intention at this stage is to provide a functional description. However, this description may encapsulate distinct mechanisms by which responses to reafferent motion are suppressed. The additional parameter could represent the gating of a visuomotor response via stronger inhibitory connections between neural units that drive pursuit and neural units that are tuned to optic flow in the reafferent direction and high contrasts. The fits of the gating model (accounting for high-contrast gating in addition to response gain modulation and contrast gain modulation) appear in colour in figure 3A,B for each perturbation condition, and in table 1. We can see that the gating model accounts well for high-contrast suppression obtained in the condition ‘Against pursuit’ (figure 3A, red line).

**Table 1.** Parameters of the gating and Naka–Rushton models fitted to the average group data.

	gating model				Naka–Rushton model		
	*R*_*max*_	*c*_*50*_	*n*	*s*	*R*_*max*_	*c*_*50*_	*n*
With pursuit	3.33	0.03	1.25	0.00	3.32	0.03	1.26
Against pursuit	1.75	0.03	2.19	6.85	0.56	0.01	10.00
Upwards	1.71	0.02	2.02	0.00	1.71	0.02	2.02
Downwards	1.79	0.02	1.28	0.35	1.43	0.02	2.25

**Figure 4.**
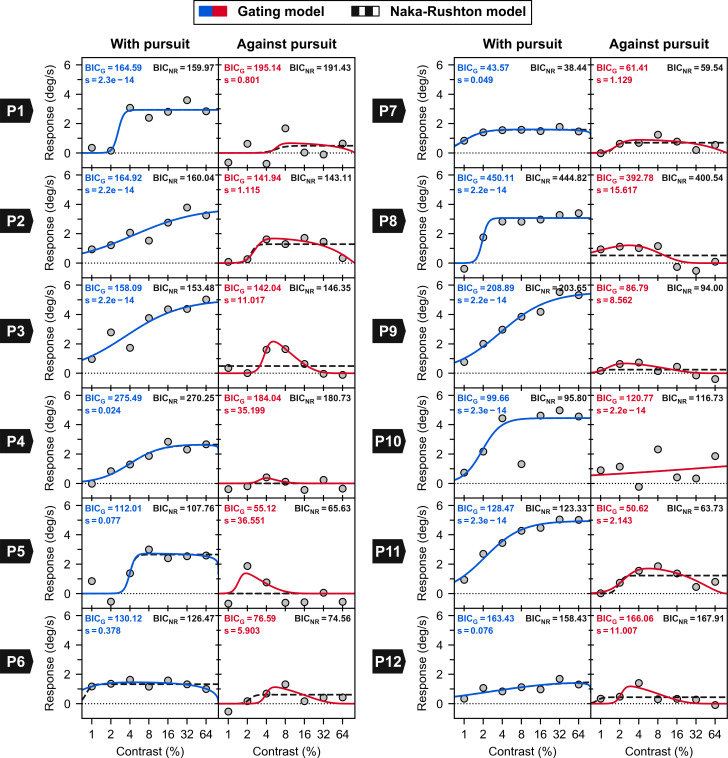
Fits of the gating and Naka–Rushton models to the data of the 12 participants for conditions with horizontal perturbation (i.e. With and Against pursuit). Coloured lines correspond to the gating model, while dashed black lines correspond to the Naka–Rushton model. BIC values are presented at the top of each panel. Note that lower BIC values indicate a better model. The fitting procedure was applied to all individual data points (all valid trials at each contrast), rather than to the means of all trials at each contrast. For clarity, those individual data points are not shown in the figure.

**Figure 5.**
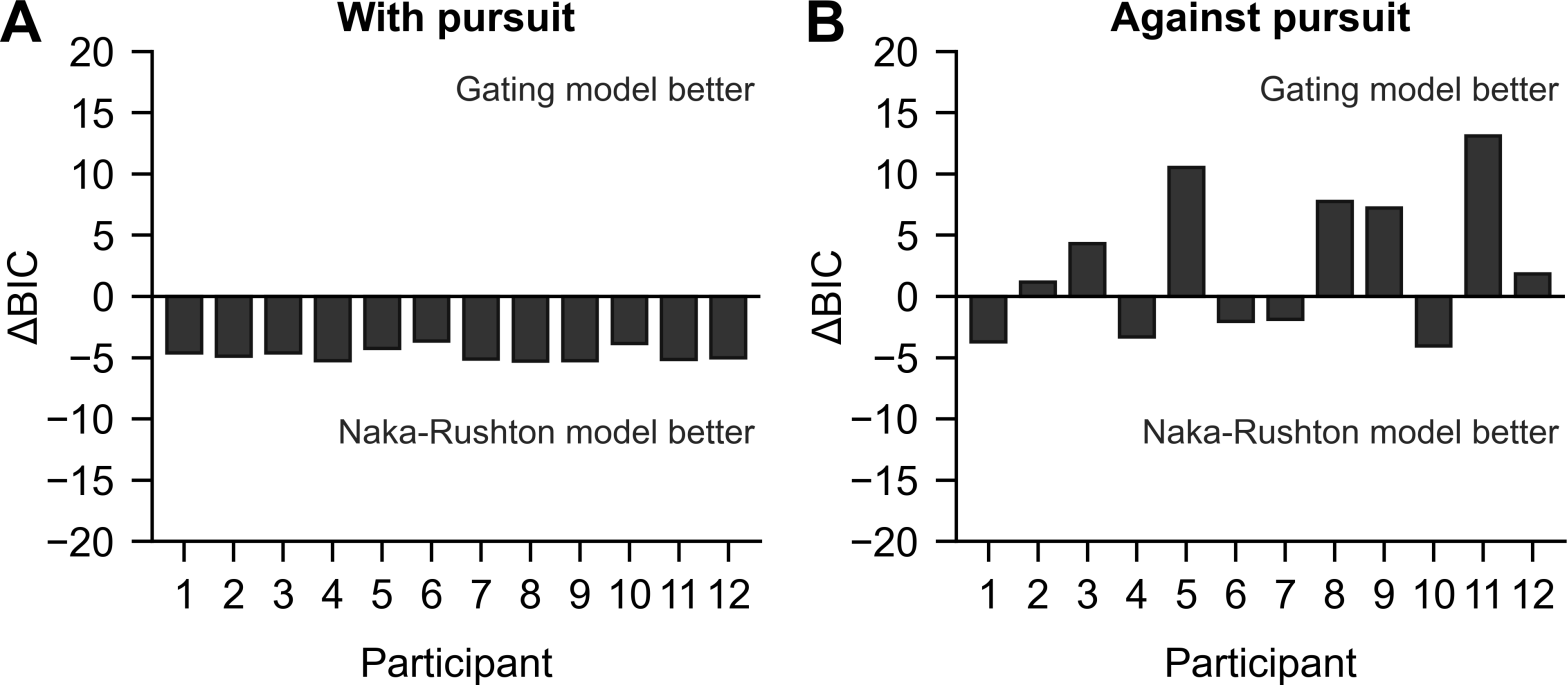
The difference between the BICs of the Naka–Rushton and gating model fit to the data of each participant from figure 4 (ΔBIC = BIC_NR_ − BIC_Gating_). (A) BIC difference for the condition ‘With pursuit’. (B) BIC difference for the condition ‘Against pursuit’. Positive values indicate that the gating model is better.

The new model is better at the individual level as well. All participants show a similar pattern of responses in the reafferent direction (‘With pursuit’), which we confirmed by comparing the Bayesian information criterion (BIC) based on the sum of squared residuals (SSR) (BIC = *k*ln(*n*) + *n*ln(*SSR/n*), where *n* is the number of observations and *k* is the number of parameters. Lower BIC values indicate a better model). Figure 4 shows the fit of the Naka–Rushton model and the gating model to the data of each participant, while table 2 contains the fitted parameters of the gating model to the individual data, and table 3 contains the parameters of the Naka–Rushton model. Starting with the condition ‘With pursuit’, the Naka–Rushton function appears as the best model for all participants. This is because, as we can see in figure 4 (dashed lines and solid lines representing Naka–Rushton and gating-model fits, respectively), the fits for this condition are virtually the same in both models, but the BIC penalizes the gating model as it has one more parameter. In that condition, the suppression parameter is very close to zero in all participants. On the other hand, in the condition ‘Against pursuit’, the gating model is superior for most participants. A close inspection of the fits in figure 4 reveals that the suppression parameter is needed to better account for the suppression at higher contrasts, which is also shown by the comparison between the BICs of each model displayed in figure 5*.* The vertical axes in this figure show the difference between the BIC of the Naka–Rushton and gating model fits. As can be seen in panel A, the Naka–Rushton is the better model for the condition ‘With pursuit’, while, as can be seen in panel B, the BIC difference heavily favours the gating model in the condition ‘Against pursuit’.

**Table 2.** Parameters of the gating model fitted to the individual data.

	With pursuit				Against pursuit			
	*R*_*max*_	*c*_*50*_	*n*	*s*	*R*_*max*_	*c*_*50*_	*n*	*s*
P1	2.94	0.03	10.00	0.00	0.73	0.06	8.88	0.80
P2	3.78	0.05	0.84	0.00	1.80	0.03	6.34	1.11
P3	5.03	0.03	0.97	0.00	4.16	0.04	8.44	11.02
P4	2.70	0.04	1.75	0.02	1.80	0.03	8.70	35.20
P5	2.74	0.04	10.00	0.08	3.00	0.01	9.94	36.55
P6	1.49	0.01	2.24	0.38	1.63	0.04	10.00	5.90
P7	1.61	0.01	2.64	0.05	0.95	0.02	5.40	1.13
P8	3.07	0.02	10.00	0.00	3.40	0.02	1.02	15.62
P9	5.54	0.04	1.14	0.00	0.87	0.01	4.74	8.56
P10	4.45	0.02	2.89	0.00	2.35	1.00	0.24	0.00
P11	4.95	0.02	1.49	0.00	2.00	0.02	3.66	2.14
P12	1.70	0.03	0.66	0.08	1.70	0.02	10.00	11.01

**Table 3.** Parameters of the Naka–Rushton model fitted to the individual data.

	With pursuit			Against pursuit		
	*R*_*max*_	*c*_*50*_	*n*	*R*_*max*_	*c*_*50*_	*n*
P1	2.94	0.03	10.00	0.49	0.06	5.31
P2	3.78	0.05	0.84	1.29	0.02	10.00
P3	5.03	0.03	0.97	0.98	0.58	0.00
P4	2.65	0.04	1.78	0.00	1.00	10.00
P5	2.65	0.04	10.00	0.00	0.01	0.00
P6	1.33	0.01	10.00	0.61	0.02	10.00
P7	1.59	0.01	2.88	0.70	0.02	10.00
P8	3.07	0.02	10.00	1.04	1.00	0.00
P9	5.54	0.04	1.14	0.24	0.01	10.00
P10	4.45	0.02	2.89	2.35	1.00	0.24
P11	4.95	0.02	1.49	1.22	0.02	10.00
P12	1.54	0.02	0.74	0.44	0.01	10.00

**Figure 6.**
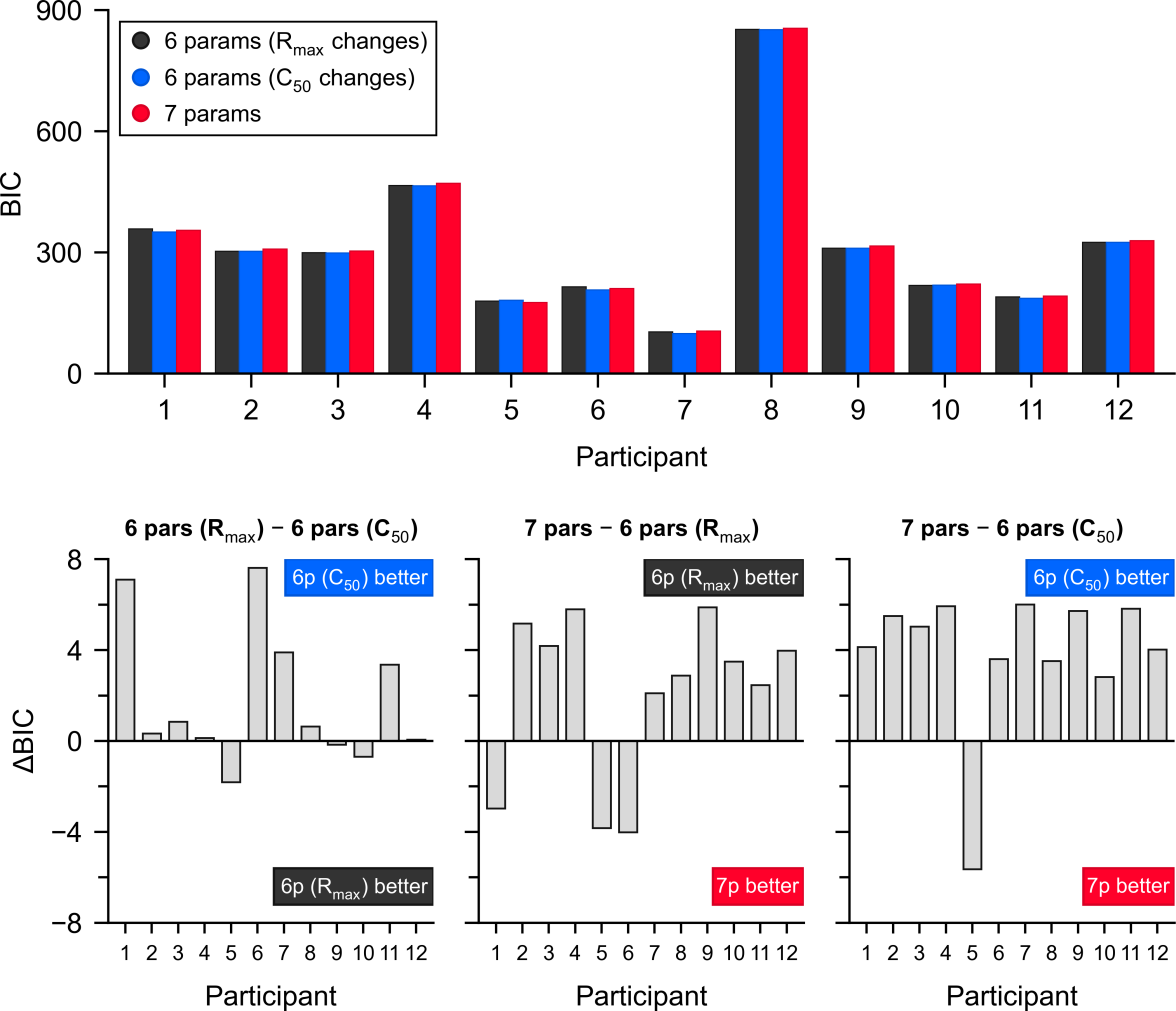
Difference in the goodness of fit according to BIC for three models fitting ‘With pursuit’ and ‘Against pursuit’ responses to background motion. A full model (seven parameters) in which both *R*_max_ and *c*_50_ can freely vary between conditions is shown in red. A more parsimonious, six-parameter model, where one *R*_max_ is shared between conditions—allowing only sensory attenuation and contrast gating—is shown in blue. Finally, a six-parameter model, where one *c*_50_ parameter is shared between conditions—allowing only response gain modulation and contrast gating—is shown in black. The upper panel shows BIC values for all participants. The lower panels show pairwise contrasts between models. Positive or negative values indicate which model is favoured per participant. Note that the best model has smaller BIC values.

After fitting the gating model to the data of each condition tested, we can now assess whether sensory attenuation (contrast gain modulation) contributes to the suppression of optokinesis in addition to response gain modulation. If the fits show a decrease of *R*_max_ between conditions, while *c*_50_ remains constant, then this would suggest that a modulation of visuomotor gain could be responsible for the effect observed. On the contrary, an increase of *c*_50_, while *R*_max_ remains stable, would suggest that the suppression of optokinesis occurs because of sensory attenuation. Because there is a necessary extra parameter, *s*, in our model, we cannot directly compare *c*_50_ or *R*_max_ between ‘With pursuit’ and ‘Against pursuit’ to decide about which parameters are responsible for the suppression, since those co-vary with *s*. Instead, we compared the BICs for three models: a six-parameter model in which only *R*_max_ and *s* are allowed to vary between conditions, but there is only one half-saturation (*c*_50_) parameter and *n* (slope) parameter—meaning that we fit different *R*_max_ and *s* for ‘Against pursuit’ and ‘With pursuit’ conditions, with a common *c*_50_ and *n*; another six-parameter model in which *c*_50_ and *s* are allowed to vary between conditions, while there are common *R*_max_ and *n* for both conditions; and a seven-parameter model where *c*_50_ is also allowed to vary between conditions—meaning that we fit three parameters for ‘Against pursuit’ (*R*_max_, *s*, *c*_50_) and three parameters for ‘With pursuit’ (*R*_max_, *s*, *c*_50_), with a common *n*. The difference between the BIC values of both models is shown in figure 6, while the fits and BIC values of each model appear in electronic supplementary materials, figures S1–S3 and tables S1–S3.

On the bottom panels of figure 6, the BIC values for the more parsimonious models (with six parameters) indicate that, for most participants (9 out of 12), the model in which only the half-saturation contrast (*c*_50_) and the high-contrast gating parameter *s* change between conditions—while *R*_max_ is shared—provides the best fit. Both parsimonious models outperform the full model, where both *R*_max_ and *c*_50_ are allowed to vary across conditions. Notably, the model allowing changes in sensory attenuation offers superior fits for the majority of the participants. Overall, these results suggest that a combination of sensory attenuation and high-contrast gating sufficiently accounts for the differences in responses between the ‘With pursuit’ and ‘Against pursuit’ conditions in most participants.

## 4. Discussion

This study aimed to differentiate between visuomotor response gain and contrast gain accounts of optokinesis suppression during smooth pursuit eye movements. As illustrated in figure 1, our initial prediction was that a modulation of response gain would occur, that is, a reduction of the asymptote but no change in the half-saturation contrast, whereas a contrast gain modulation, indicative of the attenuation of sensory inputs, would predict a rightward shift in contrast–response functions—we would need to increase the input contrast to achieve the same response. Our findings suggest that neither account fully explains the observed reflexive eye movements in the reafferent direction. To adequately fit these responses, we introduced an additional parameter that corresponds to an additional suppression: a high-contrast gating mechanism which could ensure undisturbed pursuit of a signal in the presence of a highly salient background.

Key observations:

(1) Ocular tracking responses in the perturbation direction were much larger in the pursuit direction compared with the vertical direction. This modulation maps well in a simple response gain modulation, as suggested earlier [35].
(2) Our results suggest that a third, newly observed, mechanism is necessary to explain the suppression of optokinesis as probed by background motion, amounting to a cancellation of responses towards high contrast background motion opposite to the pursuit direction.
(3) Our results suggest that the suppression of response to background motion can be explained in most individual cases by a combination of sensory attenuation and high-contrast gating or cancellation, since suppression is maximum at the highest contrasts. With the caveat that visuomotor gain downregulation could be present but masked by high-contrast gating.

Several lines of evidence suggest that visuomotor gain modulation mechanisms play a critical role in regulating how pursuit eye movements adjust in response to visual input. A higher visuomotor gain during pursuit, compared with fixation, is essential for maintaining stable tracking when faced with small retinal error signals. Single-cell recordings indicate that visuomotor gain—like the visuomotor transformation observed in saccadic eye movements—can be modulated by the frontal eye fields (FEF) [36]. FEF may deliver a top-down signal that selects targets for pursuit eye movements and increases visuomotor gain for signals in the direction of pursuit [36]. This modulation could explain several observed phenomena, including larger responses to target and background perturbations relative to fixation [35], larger responses in the pursuit direction relative to the vertical direction [35] and the suppression of optokinesis [36].

In addition to a modulation of visuomotor gain, we observe a high-contrast gating of ocular responses to background motion in the direction of reafference, which could arise as an adaption to prevailing contrasts under natural conditions. This type of adaptive suppression would need to happen in visual areas that integrate extra-retinal signals feeding information about eye movement. The exact level at which a high-contrast gating mechanism may operate remains to be elucidated. Supersaturation, observed in some neuronal responses, where maximal contrasts do not generate maximal responses, may play a role [37]. Contrast nonlinearities can have a function in detecting visual features such as conjunctions. These nonlinearities are much more common at the neuronal level in the cat and macaque cortex than previously thought. Neural contrast responses are also believed to be an adaptation to natural image statistics [38].

In the presence of supersaturating responses to contrast, we can imagine high-contrast gating as arising from adaptive suppression to the contrasts that are prevalent in the background of natural images. An agent [39] that would be rewarded by a sharper image when tracking a small target over a cluttered background would need to suppress eye movements in response to the reafferent retinal flow, as it would move away from the object of interest, resulting in a blurrier image. The reafferent retinal flow is global, unidirectional and uniform, as the rotation is opposite and of equal magnitude to the eye movement velocity. This facilitates selective visuomotor suppression of responses in the reafferent direction. If these responses are driven by motion signals carried by neurons with supersaturating responses to contrast, the backpropagation of error would suppress response to the prevalent scene contrasts but spare response to units that have maximal responses at lower contrasts. Natural image statistics suggest that while very low contrasts can occur over small patches, they are much less represented over large ones [40]. The global optic flow generated by pursuit eye movements, which processing could be suppressed in the reafferent direction, can be represented by neuronal units with large receptive fields, such as those found in the MST [41].

Whereas Lindner & Ilg [18] refer to the suppression of optokinesis when measuring reflexive responses to the background, others have measured the optokinetic response (oscillating quick and slow eye movement beat) elicited after a period of stimulation [42–44]. Further studies could test whether these two paradigms show a consistent set of mechanisms.

Our third key finding is that sensory attenuation could have a role in the suppression of oculomotor responses to background motion in the direction of reafference. Both the rightward shift in responses with contrast and the high-contrast gating mechanism could be indicative of sensory attenuation and contribute to the suppression of optokinesis during pursuit. Sensory attenuation in the reafferent direction during pursuit has been shown strongly in the form of a reduction of motion integration [16]. Sensory attenuation is present in fixational [6] and saccadic eye movements (saccadic suppression [45]) and is ubiquitous across sensory modalities, such as in processing touch [46] and speech signals [47]. It is also ubiquitous and across species, for instance, in auditory neuron’s response to self-generated song in birds [3]. The attenuation of expected signals can participate in efficient coding of sensory information, whereby computing resources are preferentially allocated to novelty [48–51]. These expectations can concern stimulus characteristics in interaction with the characteristics of goal-oriented behaviour, including how visual stimulation is shaped by the constant eye movements we make [52,53]—in our case, the global optic flow pattern generated by background features during pursuit eye movements.

While sensory attenuation is clear across the senses and in saccadic eye movements, one advantage of suppressing optokinesis without relying entirely on sensory attenuation of reafference is that the processing of reafferent signals can have an important role in recalibrating motion perception in spatial coordinates [11,12,54]. It is true that recalibration would be possible on the basis of a partially attenuated signal, but, unlike saccadic eye movements, where suppression (or omission) of reafferent information is believed to be beneficial to visual stability, the organism can benefit from awareness of peripheral events during pursuit. In addition, while very brief visual suppression during saccades is unlikely to hinder detection of important background events, strong attenuation during seconds of pursuit could pose a greater challenge.

The visual system may have found during pursuit a middle-way between omitting reafferent information and suppressing responses to reafferent information by processing reafferent information differently. Indeed, the processing of reafferent signals does not have to involve as many computational resources as external (‘exafferent’ [2]) signals. If the main use of reafferent motion processing is to provide a consolidated estimate of eye movement velocity, this estimate can be extracted by averaging only a subsample of reafferent signals, given their uniformity. We would then expect sensory attenuation in the integration of complex motion signals, as required, for instance, to extract an object’s direction based on the motion of its local contours but not when a simple averaging is required [55,56].

In summary, we show that the suppression of reflexive oculomotor responses to background motion (optokinesis) in the reafferent direction during pursuit eye movements does not conform to a simple response gain modulation, contrast gain modulation or any combination of contrast and response gain modulation, but instead it includes a high-contrast gating mechanism, which could have arisen through adaptation to natural image statistics.

## Data Availability

Code used for stimulus display, data analysis and modelling is available on the Open Science Framework website [57]. Supplementary material is available online [58].

## References

[1] Sperry RW. 1950 Neural basis of the spontaneous optokinetic response produced by visual inversion. J. Comp. Physiol. Psychol. **43**, 482–489. (10.1037/h0055479) 14794830

[2] von Holst E, Mittelstaedt H. 1950 Das reafferenzprinzip. Naturwissenschaften **37**, 464–476. (10.1007/BF00622503)

[3] Crapse TB, Sommer MA. 2008 Corollary discharge across the animal kingdom. Nat. Rev. Neurosci. **9**, 587–600. (10.1038/nrn2457) 18641666 PMC5153363

[4] Sommer MA, Wurtz RH. 2008 Visual perception and corollary discharge. Perception **37**, 408–418. (10.1068/p5873) 18491718 PMC2807735

[5] Binda P, Morrone MC. 2018 Vision during saccadic eye movements. Annu. Rev. Vis. Sci. **4**, 193–213. (10.1146/annurev-vision-091517-034317) 30222534

[6] Scholes C, McGraw PV, Roach NW. 2021 Learning to silence saccadic suppression. Proc. Natl Acad. Sci. USA **118**, e2012937118. (10.1073/pnas.2012937118) 33526665 PMC8018005

[7] Castet E, Jeanjean S, Masson GS. 2002 Motion perception of saccade-induced retinal translation. Proc. Natl Acad. Sci. USA **99**, 15159–15163. (10.1073/pnas.232377199) 12417765 PMC137560

[8] Shioiri S, Cavanagh P. 1989 Saccadic suppression of low-level motion. Vision Res. **29**, 915–928. (10.1016/0042-6989(89)90106-5) 2629207

[9] Frost A, Niemeier M. 2015 Suppression and reversal of motion perception around the time of the saccade. Front. Syst. Neurosci. **9**, 143. (10.3389/fnsys.2015.00143) 26582270 PMC4628122

[10] Goettker A, Braun DI, Schütz AC, Gegenfurtner KR. 2018 Execution of saccadic eye movements affects speed perception. Proc. Natl Acad. Sci. USA **115**, 2240–2245. (10.1073/pnas.1704799115) 29440494 PMC5834663

[11] Haarmeier T, Thier P, Repnow M, Petersen D. 1997 False perception of motion in a patient who cannot compensate for eye movements. Nature **389**, 849–852. (10.1038/39872) 9349816

[12] Haarmeier T, Bunjes F, Lindner A, Berret E, Thier P. 2001 Optimizing visual motion perception during eye movements. Neuron **32**, 527–535. (10.1016/s0896-6273(01)00486-x) 11709162

[13] Filehne W. 1922 Uber das optische Wahrnehmen von Bewegungen. Z. Fur Sinnephysiologie **53**, 134–145.

[14] Brenner E, van den Berg AV. 1994 Judging object velocity during smooth pursuit eye movements. Exp. Brain Res. **99**, 316–324. (10.1007/BF00239598) 7925812

[15] Freeman TCA, Champion RA, Warren PA. 2010 A Bayesian model of perceived head-centered velocity during smooth pursuit eye movement. Curr. Biol. **20**, 757–762. (10.1016/j.cub.2010.02.059) 20399096 PMC2861164

[16] Souto D, Chudasama J, Kerzel D, Johnston A. 2019 Motion integration is anisotropic during smooth pursuit eye movements. J. Neurophysiol. **121**, 1787–1797. (10.1152/jn.00591.2018) 30840536 PMC6589720

[17] Lindner A, Schwarz U, Ilg UJ. 2001 Cancellation of self-induced retinal image motion during smooth pursuit eye movements. Vision Res. **41**, 1685–1694. (10.1016/s0042-6989(01)00050-5) 11348650

[18] Lindner A, Ilg UJ. 2006 Suppression of optokinesis during smooth pursuit eye movements revisited: the role of extra-retinal information. Vision Res. **46**, 761–767. (10.1016/j.visres.2005.09.033) 16274723

[19] Naka KI, Rushton WAH. 1966 S‐potentials from colour units in the retina of fish (Cyprinidae). J. Physiol. **185**, 536–555. (10.1113/jphysiol.1966.sp008001) 5918058 PMC1395833

[20] Barthélemy FV, Perrinet LU, Castet E, Masson GS. 2008 Dynamics of distributed 1D and 2D motion representations for short-latency ocular following. Vision Res. **48**, 501–522. (10.1016/j.visres.2007.10.020) 18221979

[21] Lisberger SG. 1998 Postsaccadic enhancement of initiation of smooth pursuit eye movements in monkeys. J. Neurophysiol. **79**, 1918–1930. (10.1152/jn.1998.79.4.1918) 9535958

[22] Lisberger SG. 2010 Visual guidance of smooth-pursuit eye movements: sensation, action, and what happens in between. Neuron **66**, 477–491. (10.1016/j.neuron.2010.03.027) 20510853 PMC2887486

[23] Tanaka M, Lisberger SG. 2001 Regulation of the gain of visually guided smooth-pursuit eye movements by frontal cortex. Nature **409**, 191–194. (10.1038/35051582) 11196642

[24] Suehiro K, Miura KI, Kodaka Y, Inoue Y, Takemura A, Kawano K. 1999 Effects of smooth pursuit eye movement on ocular responses to sudden background motion in humans. Neurosci. Res. **35**, 329–338. (10.1016/s0168-0102(99)00098-x) 10617324

[25] Kodaka Y, Miura K, Suehiro K, Takemura A, Kawano K. 2004 Ocular tracking of moving targets: effects of perturbing the background. J. Neurophysiol. **91**, 2474–2483. (10.1152/jn.01079.2003) 14762158

[26] Schwarz U, Ilg UJ. 1999 Asymmetry in visual motion processing. NeuroReport **10**, 2477–2480. (10.1097/00001756-199908200-00008) 10574355

[27] Ilg UJ. 2008 The role of areas MT and MST in coding of visual motion underlying the execution of smooth pursuit. Vision Res. **48**, 2062–2069. (10.1016/j.visres.2008.04.015) 18508104

[28] Schütz AC, Delipetkos E, Braun DI, Kerzel D, Gegenfurtner KR. 2007 Temporal contrast sensitivity during smooth pursuit eye movements. J. Vis. **7**, 3. (10.1167/7.13.3) 17997631

[29] Tong J, Patel SS, Bedell HE. 2005 Asymmetry of perceived motion smear during head and eye movements: evidence for a dichotomous neural categorization of retinal image motion. Vision Res. **45**, 1519–1524. (10.1016/j.visres.2004.12.004) 15781070

[30] Tong J, Patel SS, Bedell HE. 2006 The attenuation of perceived motion smear during combined eye and head movements. Vision Res. **46**, 4387–4397. (10.1016/j.visres.2006.08.034) 17046046 PMC1752240

[31] Brainard DH. 1997 The psychophysics toolbox. Spat. Vis. **10**, 433–436. (10.1163/156856897x00357) 9176952

[32] Kleiner M, Brainard D, Pelli D, Ingling A, Murray R, Broussard C. 2007 What’s new in Psychtoolbox-3? Perception **36**, 1–16.

[33] Miura K, Kobayashi Y, Kawano K. 2009 Ocular responses to brief motion of textured backgrounds during smooth pursuit in humans. J. Neurophysiol. **102**, 1736–1747. (10.1152/jn.00430.2009) 19605610

[34] Bahill AT, McDonald JD. 1983 Frequency limitations and optimal step size for the two-point central difference derivative algorithm with applications to human eye movement data. IEEE Trans. Biomed. Eng. **30**, 191–194. (10.1109/tbme.1983.325108) 6862495

[35] Churchland AK, Lisberger SG. 2002 Gain control in human smooth-pursuit eye movements. J. Neurophysiol. **87**, 2936–2945. (10.1152/jn.2002.87.6.2936) 12037197 PMC2582523

[36] Izawa Y, Suzuki H. 2020 Suppressive control of optokinetic and vestibular nystagmus by the primate frontal eye field. J. Neurophysiol. **124**, 691–702. (10.1152/jn.00015.2020) 32727256

[37] Peirce JW. 2007 The potential importance of saturating and supersaturating contrast response functions in visual cortex. J. Vis. **7**, 13. (10.1167/7.6.13) PMC2082665 17685796

[38] Clatworthy PL, Chirimuuta M, Lauritzen JS, Tolhurst DJ. 2003 Coding of the contrasts in natural images by populations of neurons in primary visual cortex (V1). Vision Res. **43**, 1983–2001. (10.1016/s0042-6989(03)00277-3) 12831760

[39] Eckmann S, Klimmasch L, Shi BE, Triesch J. 2020 Active efficient coding explains the development of binocular vision and its failure in amblyopia. Proc. Natl Acad. Sci. USA **117**, 6156–6162. (10.1073/pnas.1908100117) 32123102 PMC7084066

[40] Frazor RA, Geisler WS. 2006 Local luminance and contrast in natural images. Vision Res. **46**, 1585–1598. (10.1016/j.visres.2005.06.038) 16403546

[41] Duffy CJ, Wurtz RH. 1997 Multiple temporal components of optic flow responses in MST neurons. Exp. Brain Res. **114**, 472–482. (10.1007/pl00005656) 9187283

[42] Pola J, Wyatt HJ, Lustgarten M. 1992 Suppression of optokinesis by a stabilized target: effects of instruction and stimulus frequency. Percept. Psychophys. **52**, 186–200. (10.3758/bf03206772) 1508626

[43] Pola J, Wyatt HJ, Lustgarten M. 1995 Visual fixation of a target and suppression of optokinetic nystagmus: effects of varying target feedback. Vision Res. **35**, 1079–1087. (10.1016/0042-6989(94)00215-8) 7762164

[44] Wyatt HJ, Pola J. 1984 A mechanism for suppression of optokinesis. Vision Res. **24**, 1931–1945. (10.1016/0042-6989(84)90027-0) 6534017

[45] Krekelberg B. 2010 Saccadic suppression. Curr. Biol. **20**, R228–R229. (10.1016/j.cub.2009.12.018) 20219169

[46] Blakemore SJ, Wolpert DM, Frith CD. 1998 Central cancellation of self-produced tickle sensation. Nat. Neurosci. **1**, 635–640. (10.1038/2870) 10196573

[47] Kiepe F, Kraus N, Hesselmann G. 2021 Sensory attenuation in the auditory modality as a window into predictive processing. Front. Hum. Neurosci. **15**, 704668. (10.3389/fnhum.2021.704668) 34803629 PMC8602204

[48] Festa D, Aschner A, Davila A, Kohn A, Coen-Cagli R. 2021 Neuronal variability reflects probabilistic inference tuned to natural image statistics. Nat. Commun. **12**, 3635. (10.1038/s41467-021-23838-x) 34131142 PMC8206154

[49] Olshausen BA, Field DJ. 1996 Emergence of simple-cell receptive field properties by learning a sparse code for natural images. Nature **381**, 607–609. (10.1038/381607a0) 8637596

[50] Barlow HB. 1961 Possible principles underlying the transformations of sensory messages. In Sensory communication (ed. WA Rosenblith), pp. 217–234. Cambridge, MA: MIT Press. (10.7551/mitpress/9780262518420.003.0013)

[51] Attneave F. 1954 Some informational aspects of visual perception. Psychol. Rev. **61**, 183–193. (10.1037/h0054663) 13167245

[52] Rolfs M, Schweitzer R. 2022 Coupling perception to action through incidental sensory consequences of motor behaviour. Nat. Rev. Psychol. **1**, 112–123. (10.1038/s44159-021-00015-x)

[53] Turner MH, Sanchez Giraldo LG, Schwartz O, Rieke F. 2019 Stimulus- and goal-oriented frameworks for understanding natural vision. Nat. Neurosci. **22**, 15–24. (10.1038/s41593-018-0284-0) 30531846 PMC8378293

[54] Luna R, Serrano-Pedraza I, Gegenfurtner KR, Schütz AC, Souto D. 2021 Achieving visual stability during smooth pursuit eye movements: directional and confidence judgements favor a recalibration model. Vision Res. **184**, 58–73. (10.1016/j.visres.2021.03.003) 33873123

[55] Ovsepian R, Souto D, Schütz AC. 2025 Robust generalization of tuning to self-induced sensation. iScience **28**, 112563. (10.1016/j.isci.2025.112563) 40487431 PMC12144454

[56] Schütz AC, Ovsepian R, Souto D. 2025 Self-induced motion is recalibrated based on the vector average during smooth pursuit eye movements. [Abstract]. In Presented at the 48th European Conference on Visual Perception (ECVP), pp. 24–28. Mainz, Germany.

[57] Souto D, Bachtoula O. 2025 Multiple Mechanisms of Response Suppression to Self-induced Sensation During Pursuit Eye Movements https://osf.io/5jm2r/overview 10.1098/rsos.250967 PMC12567075 41164333

[58] Bachtoula, O, Ellul Mel M, Serrano-Pedraza I, Souto D. 2025 Supplementary material from: Multiple mechanisms of response suppression to self-induced sensation during pursuit eye movements. Figshare. (10.6084/m9.figshare.c.8085125) PMC12567075 41164333

